# Serum zonulin levels and their association with inflammatory and oxidative stress biomarkers in patients undergoing maintenance hemodialysis

**DOI:** 10.1016/j.jgeb.2026.100842

**Published:** 2026-10-15

**Authors:** Ali B. Roomi, Hussein Jawad Kadhim, Abbas Khairi Fadhil, Ibrahim Abdullah Hamzah

**Affiliations:** a Department of Biochemistry, College of Medicine, University of Thi-Qar, Nasiriyah, Thi-Qar, 64001, IRAQ; b Technical Engineering College, Al-Ayen Iraqi University, Nasiriyah, Thi-Qar, 64001, Iraq

**Keywords:** Hemodialysis, Zonulin, Intestinal permeability, Oxidative stress, Interleukin-6, End-stage renal disease, Gut-kidney axis

## Abstract

**Background:**

Maintenance hemodialysis (HD) is accompanied by persistent inflammation, oxidative stress, and disturbances of the intestinal barrier.

**Objective:**

To compare serum zonulin concentrations between patients receiving maintenance HD and healthy controls and to evaluate their associations with interleukin-6 (IL-6), malondialdehyde (MDA), and total antioxidant status (TAS).

**Methods:**

This comparative cross-sectional study included 180 participants: 90 patients with end-stage renal disease receiving maintenance HD and 90 age- and sex-comparable healthy controls. Predialysis blood samples were analyzed for serum zonulin, IL-6, and MDA by enzyme-linked immunosorbent assay and for TAS by a colorimetric assay. Group differences were evaluated using independent-samples *t*-tests, associations using Pearson's correlation, and adjusted relationships using multivariable linear regression.

**Results:**

Compared with controls, the HD group had higher serum zonulin, IL-6, MDA, urea, creatinine, and phosphorus concentrations and lower TAS, albumin, calcium, and hemoglobin values (all *P* < 0.001). Within the HD group, zonulin correlated positively with IL-6 (*r* = 0.644, *P* < 0.001) and MDA (*r* = 0.782, *P* < 0.001) and inversely with TAS (*r* = −0.392, *P* < 0.001). After covariate adjustment, zonulin remained associated with IL-6 (B = 0.982, β = 0.577, *P* < 0.001), MDA (B = 36.76, β = 0.597, *P* < 0.001), and TAS (B = −0.018, β = −0.345, *P* = 0.021).

**Conclusion:**

Serum zonulin was elevated in patients receiving maintenance HD and was independently associated with inflammatory and oxidative-stress biomarkers. These cross-sectional findings support an association but do not establish that altered intestinal permeability causes inflammatory-oxidative imbalance or that serum zonulin is ready for clinical use.

## 1 Introduction

Chronic kidney disease (CKD) is a major global health burden. In 2023, an estimated 788 million adults were living with CKD, and CKD accounted for approximately 1.48 million deaths, ranking ninth among causes of death worldwide.^1^ Progressive loss of kidney function may culminate in kidney failure, also termed end-stage renal disease (ESRD), for which kidney replacement therapy is required. Among patients receiving maintenance hemodialysis (HD), the burden extends beyond impaired filtration and includes persistent low-grade inflammation, oxidative imbalance, endothelial injury, metabolic disturbances, and immune dysfunction. Together, these abnormal findings are responsible for the cardiovascular morbidity and mortality seen in this patient population.^2^, ^3^, ^4^, ^5^ Despite advancements in dialysis techniques and supportive care, inflammation and oxidative stress are still important non-traditional risk factors for adverse events.^4^, ^6^. In addition, the process of hemodialysis itself might activate inflammation due to repetitive contact of blood with the dialysis membrane, complement activation, oxidative damage, endotoxin presence, and biocompatibility-induced immune response.^7^, ^8^ Interleukin-6 (IL-6) has special importance, since elevated levels of IL-6 in ESRD patients have been linked to chronic microinflammation, protein-energy wasting, endothelial dysfunction, cardiovascular diseases, and mortality.^9^, ^10^, ^11^, ^12^ Oxidative stress, which can be seen as an imbalance between the formation of reactive oxygen species and antioxidant protection, is yet another crucial factor of CKD.^13^, ^14^ Elevated lipoperoxidation, usually measured via MDA, along with decreased TAS, has been demonstrated in patients undergoing HD therapy.^13^, ^14^, ^15^, ^16^

It is possible that the gut-kidney axis could play a role in such systemic alterations. In patients with CKD, gut dysbiosis is accompanied by alteration in tight junctions and leaky gut. The resulting impairment in the barrier structure may enable transport of products from bacteria and gut microbiota into the blood, contributing to systemic inflammation.^17^, ^18^, ^19^, ^20^, ^21^ Uremic toxic load, oxidative stress, and immune dysfunction are all features of maintenance HD along with impairment of the gut barrier function, while alterations in the microbiota composition have been linked to inflammation, cardiovascular disease, protein energy wasting, and immune disorders.^19^, ^21^, ^22^, ^23^

Zonulin is involved in regulating the permeability of epithelial tight junctions and is commonly employed as an indirect biomarker for disruption of the intestinal barrier.^24^, ^25^ However, commercially available zonulin serum tests may measure zonulin-associated proteins other than pre-haptoglobin-2, and serum levels of the protein do not necessarily correlate with intestinal permeability.^26^, ^27^

Serum zonulin should therefore be interpreted as a putative, assay-dependent research marker rather than a definitive measure of gut permeability. Higher concentrations have been reported in inflammatory, immune-mediated, and metabolic disorders,^28^, ^29^ whereas studies in CKD and other renal diseases have produced mixed results regarding associations with renal dysfunction, systemic inflammation, endothelial impairment, and disease-specific outcomes.^30^, ^31^, ^32^, ^33^ Experimental and clinical evidence suggests that impaired intestinal barrier integrity may coexist with endotoxemia, inflammatory signaling, and oxidative stress.^34^ However, the relationship between serum zonulin and paired inflammatory, lipid-peroxidation, and antioxidant markers in maintenance HD remains insufficiently characterized. Accordingly, this study compared serum zonulin, IL-6, MDA, and TAS between patients receiving maintenance HD and healthy controls and evaluated whether serum zonulin was associated with IL-6, MDA, and TAS after adjustment for selected demographic and clinical covariates.

## 2 Materials and methods

### 2.1 Study design

This single-center comparative cross-sectional study included 180 participants: 90 adults with ESRD receiving maintenance HD and 90 healthy controls comparable in age and sex. Patients were recruited from the Hemodialysis Unit at Al-Hussein Teaching Hospital, Thi-Qar Health Directorate, Nasiriyah, Iraq. Blood samples from the HD group were collected immediately before a scheduled dialysis session to reduce the influence of intradialytic biochemical shifts on the biomarkers under investigation.

### 2.2 Participants and eligibility criteria

Eligible patients were adults receiving regular maintenance HD. Controls were assessed through medical history and were required to have no known CKD, inflammatory or autoimmune disease, liver disease, malignancy, or acute infection at the time of sampling. In both groups, individuals with active infection, chronic inflammatory or autoimmune disease, malignancy, severe hepatic dysfunction, or another major systemic illness likely to influence inflammatory or oxidative-stress biomarkers were excluded. The available study records did not preserve a more granular classification of the control recruitment source, such as hospital staff, community volunteers, or patients' relatives.

### 2.3 Blood sampling and serum preparation

Blood was collected under aseptic conditions. For the HD group, sampling was performed before the dialysis session. Approximately 10 mL of venous blood was collected into ethylenediaminetetraacetic acid-containing and plain tubes, according to the requirements of the planned laboratory tests. Blood in plain tubes was allowed to clot, and serum was separated by centrifugation at 3000 rpm for 10 min and aliquoted into sterile microtubes. Serum samples were stored at −20 °C until biochemical and immunological analysis, and repeated freeze-thaw cycles were minimized.

### 2.4 Laboratory measurements

Serum zonulin, IL-6, and MDA were measured using commercial enzyme-linked immunosorbent assay kits (Elabscience Biotechnology Inc., Houston, TX, USA) according to the manufacturer's instructions. Sandwich formats were used for zonulin and IL-6, whereas MDA was measured using a competitive format. Absorbance was read using a STAT FAX 4200 microplate reader (Awareness Technology Inc., USA). TAS was measured with an Elabscience colorimetric kit based on the reduction of 2,2′-azino-bis(3-ethylbenzothiazoline-6-sulfonic acid) radical cations by antioxidants in the sample. Routine biochemical variables were measured using BIOLABO reagents (BIOLABO SAS, Maizy, France): urea by an ultraviolet kinetic enzymatic method, creatinine by an enzymatic colorimetric method, albumin by the bromocresol green method, calcium by the o-cresolphthalein complexone method, and phosphorus by the ultraviolet phosphomolybdate method. Hemoglobin was measured using a Mindray BC-10 hematology analyzer (Mindray, China).

### 2.5 Statistical analysis

Statistical analyses were performed using JASP version 0.18.3. Distributional normality was assessed with the Shapiro-Wilk test, and continuous variables with approximately normal distributions were summarized as mean ± standard deviation. Between-group differences were evaluated using independent-samples *t*-tests for continuous variables and chi-square tests for categorical variables. Pearson's correlation coefficient (r) was retained from the original analysis.

The archived analysis output for this revision did not include diagnostic plots for linearity or influential observations, which limitation is stated in the Study Limitations section. The effect sizes obtained from Cohen's d were standardized between-group differences and were evaluated based on conventional cut-off values of 0.2, 0.5, and 0.8 for small, medium, and large effect sizes, respectively. Separate multivariable linear regression models for IL-6, MDA, and TAS were done within the HD group. The predictor variable used was the serum zonulin level. Demographic covariates included age and sex, while the clinical covariates included diabetes, hypertension, and smoking. Albumin, hemoglobin, and phosphorus were the indicators used for nutrition, hematology, and mineral metabolism, respectively.

Results are reported as unstandardized coefficients (B), standardized coefficients (β), 95% confidence intervals (CIs), and exact *P* values. Variance inflation factors (VIFs) below 5 were taken to indicate no material multicollinearity. Statistical significance was defined by a two-sided *P* value <0.05. No formal multiplicity correction was applied; therefore, effect sizes, CIs, and the consistency of related findings were considered alongside *P* values.

## 3 Results

### 3.1 Baseline characteristics of the study population

The study included 90 patients receiving maintenance HD and 90 controls. As shown in Table 1, mean age did not differ significantly between groups (*P* = 0.118), and the sex distribution was identical. Among patients receiving HD, 43 (47.8%) had diabetes mellitus and 33 (36.7%) had hypertension. Current smoking did not differ significantly between groups (*P* = 0.411). Diabetes, hypertension, and smoking were subsequently considered in the adjusted analyses because of their potential relationships with inflammatory and oxidative-stress biomarkers.

**Table 1:** Baseline Characteristics of the Study Population.

Variable	Controls	HD Patients	*P*-value
Age (years)	41.24 ± 16.12	44.87 ± 15.95	0.118
Sex (Male/Female)	45/45	45/45	1.000
Diabetes Mellitus, n (%)	0 (0%)	43 (47.8%)	< 0.001
Hypertension, n (%)	0 (0%)	33 (36.7%)	< 0.001
Current Smokers, n (%)	16 (17.7%)	12 (13.3%)	0.411

Comparisons were performed using independent-samples *t*-tests or chi-square tests, as appropriate. Data are presented as mean ± standard deviation or n (%). HD, hemodialysis.

### 3.2 Intestinal permeability, inflammatory, and oxidative stress biomarkers

Table 2 and Fig. 1 summarize the biochemical and biomarker differences between groups. Patients receiving HD had higher serum zonulin, IL-6, MDA, urea, creatinine, and phosphorus values and lower TAS, albumin, calcium, and hemoglobin values than controls (all *P* < 0.001). Cohen's d ranged from 1.913 for calcium to 5.570 for creatinine; thus, every standardized difference exceeded the conventional threshold for a large effect. The effects for zonulin (d = 4.070), IL-6 (d = 3.972), MDA (d = 3.336), and TAS (d = 3.169) indicate pronounced separation between the sampled groups, but they do not by themselves establish diagnostic accuracy, prognostic value, or clinical decision thresholds.

**Table 2:** Comparison of Biochemical, Inflammatory, Oxidative Stress, and Intestinal Permeability Biomarkers Between Hemodialysis Patients and Controls.

Parameter	Controls	Hemodialysis	t	Cohen's d	*P* value
Zonulin (ng/mL)	5.51 ± 1.64	19.06 ± 4.41	−27.30	4.070	< 0.001
IL-6 (pg/mL)	3.63 ± 1.53	25.15 ± 7.51	−26.65	3.972	< 0.001
MDA (ng/mL)	164.10 ± 59.88	820.20 ± 271.70	−22.38	3.336	< 0.001
TAS (Eq./L)	1.602 ± 0.215	0.905 ± 0.225	21.26	3.169	< 0.001
Urea (mg/dL)	32.72 ± 3.95	103.10 ± 24.77	−26.64	3.971	< 0.001
Creatinine (mg/dL)	0.658 ± 0.089	5.313 ± 1.179	−37.36	5.570	< 0.001
Albumin (g/dL)	4.319 ± 0.347	3.296 ± 0.352	19.64	2.929	< 0.001
Calcium (mg/dL)	9.176 ± 0.536	8.209 ± 0.473	12.83	1.913	< 0.001
Phosphorus (mg/dL)	3.688 ± 0.376	5.680 ± 1.295	−14.03	2.092	< 0.001
Hemoglobin (g/dL)	13.23 ± 1.43	9.645 ± 1.307	17.57	2.619	< 0.001

Data are presented as mean ± standard deviation. HD, hemodialysis; IL-6, interleukin-6; MDA, malondialdehyde; TAS, total antioxidant status.

**Fig. 1:**
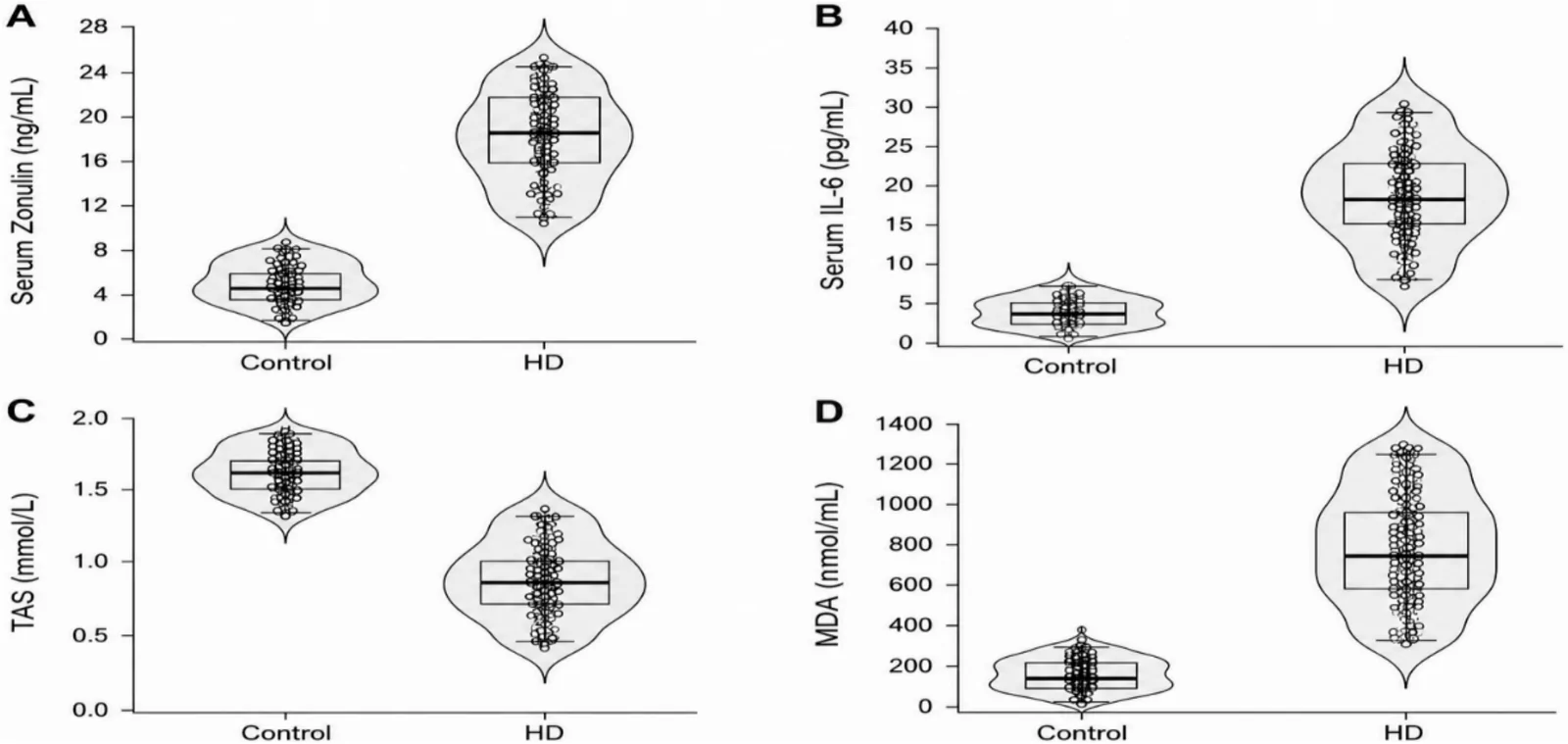
Distribution of serum zonulin, IL-6, TAS, and MDA levels in maintenance hemodialysis patients and healthy controls.

The violin graphs depict the levels of serum zonulin (A), IL-6 (B), TAS (C), and MDA (D) in HD maintenance therapy (*n* = 90) and control subjects (*n* = 90). The boxplot in the violin graph indicates the median and interquartile range while the width of the violin corresponds to the estimated distribution density. Comparison between the groups was made using independent-samples *t*-test and each biomarker was significantly different between the groups at *P* < 0.001.

### 3.3 Correlation analysis

In the case of HD patients, there were correlations between serum zonulin and IL-6 (*r* = 0.644, *P* < 0.001) as well as MDA (*r* = 0.782, *P* < 0.001) and inverse correlation with TAS (*r* = −0.392, *P* < 0.001) (Table 3). Similarly, IL-6 was positively correlated with MDA (*r* = 0.731, *P* < 0.001), whereas both IL-6 and MDA were inversely correlated with TAS (*r* = −0.674, *r* = −0.735, respectively; *P* < 0.001 for both).

**Table 3:** Pearson Correlation Analysis of Intestinal Permeability, Inflammatory, and Oxidative Stress Biomarkers in HD Patients.

Variable Pair	r	*P*-value
Zonulin vs IL-6	0.644	< 0.001
Zonulin vs MDA	0.782	< 0.001
Zonulin vs TAS	−0.392	< 0.001
IL-6 vs MDA	0.731	< 0.001
IL-6 vs TAS	−0.674	< 0.001
MDA vs TAS	−0.735	< 0.001

-The Pearson's r was kept in the original analysis after testing the distributions with the Shapiro-Wilk test. Full plots of linearity and influential observations were not available for revision and thus the results should be viewed with due care. *P* < 0.05 in two-sided tests was considered to indicate statistical significance. HD, hemodialysis; IL-6, interleukin-6; MDA, malondialdehyde; TAS, total antioxidant status.

For determination of whether the relationships seen before remained after adjustment for covariates, multivariate linear regression analysis was done separately on the HD group. As is evident from (See Table 1, Table 2, Table 3, Table 4.) Table 4, serum zonulin was found to be significantly related to IL-6, MDA, and TAS after controlling for age, gender, hypertension, diabetes mellitus, smoking, albumin, hemoglobin, and phosphorus.

**Table 1:** Baseline Characteristics of the Study Population.

Variable	Controls	HD Patients	*P*-value
Age (years)	41.24 ± 16.12	44.87 ± 15.95	0.118
Sex (Male/Female)	45/45	45/45	1.000
Diabetes Mellitus, n (%)	0 (0%)	43 (47.8%)	< 0.001
Hypertension, n (%)	0 (0%)	33 (36%)	< 0.001
Current Smokers, n (%)	16 (17.7%)	12 (13.3%)	0.411

-Comparisons between study groups were made using the independent samples (*t*-test) or (chi-squared) test. Data are shown as mean ± standard deviation (SD) or as a percentage.

**Table 2:** Comparison of Biochemical, Inflammatory, Oxidative Stress, and Intestinal Permeability Biomarkers Between Hemodialysis Patients and Controls.

Parameter	Controls	HD Patients	t-value	Cohen's d	P-value
Zonulin (ng/mL)	5.51 ± 1.64	19.06 ± 4.41	−27.30	4.070	< 0.001
IL-6 (pg/mL)	3.63 ± 1.53	25.15 ± 7.51	−26.65	3.972	< 0.001
MDA (ng/mL)	164.10 ± 59.88	820.20 ± 271.70	−22.38	3.336	< 0.001
TAS (Eq./L)	1.602 ± 0.215	0.905 ± 0.225	21.26	3.169	< 0.001
Urea (mg/dL)	32.72 ± 3.95	103.10 ± 24.77	−26.64	3.971	< 0.001
Creatinine (mg/dL)	0.658 ± 0.089	5.313 ± 1.179	−37.36	5.570	< 0.001
Albumin (g/dL)	4.319 ± 0.347	3.296 ± 0.352	19.64	2.929	< 0.001
Calcium (mg/dL)	9.176 ± 0.536	8.209 ± 0.473	12.83	1.913	< 0.001
Phosphorus (mg/dL)	3.688 ± 0.376	5.680 ± 1.295	−14.03	2.092	< 0.001
Hemoglobin (g/dL)	13.23 ± 1.43	9.645 ± 1.307	17.57	2.619	< 0.001

- mean ± standard deviation (SD); HD, hemodialysis; IL-6, interleukin-6; MDA, serum malondialdehyde; TAS, total antioxidant status.

**Table 3:** Pearson Correlation Analysis of Intestinal Permeability, Inflammatory, and Oxidative Stress Biomarkers in HD Patients.

Variable Pair	R	P-value
Zonulin vs IL-6	0.644	< 0.001
Zonulin vs MDA	0.782	< 0.001
Zonulin vs TAS	−0.392	< 0.001
IL-6 vs MDA	0.731	< 0.001
IL-6 vs TAS	−0.674	< 0.001
MDA vs TAS	−0.735	< 0.001

Abbreviations: Pearson correlation coefficient (R) was used to assess relations between values. A P-value <0.01 was considered statistically significant.

**Table 4:** Multivariable Linear Regression Analysis of the Independent Association Between Serum Zonulin and IL-6, MDA, and TAS in Hemodialysis Patients.

Dependent variable	B for zonulin	CI for B 95%	Standardized β	P-value	R^2^	Adjusted R^2^	Model P-value	VIF
IL-6	0.982	0.579–1.384	0.577	< 0.001	0.463	0.403	< 0.001	2.100
MDA	36.76	25.32–48.20	0.597	< 0.001	0.668	0.631	< 0.001	2.100
TAS	−0.018	−0.032 to −0.003	−0.345	0.021	0.184	0.092	0.049	2.100

Abbreviations: B, unstandardized regression coefficient; β, standardized beta coefficient; CI 95%, confidence interval; VIF, variance inflation factor.

**Table 4:** Multivariable Linear Regression Analysis for Adjusted Relationship Between Serum Zonulin and IL-6, MDA, and TAS Among Hemodialysis Subjects.

Dependent variable	Zonulin B	95% CI	β	*P* value	R^2^	Adjusted R^2^	Model P value	VIF
IL-6	0.982	0.579 to 1.384	0.577	< 0.001	0.463	0.403	< 0.001	2.100
MDA	36.76	25.32 to 48.20	0.597	< 0.001	0.668	0.631	< 0.001	2.100
TAS	−0.018	−0.032 to −0.003	−0.345	0.021	0.184	0.092	0.049	2.100

B, unstandardized regression coefficient; β, standardized coefficient; CI, confidence interval; VIF, variance inflation factor. The table reports the VIF for zonulin; VIF values for every predictor in each model were < 5, indicating no material multicollinearity. HD, hemodialysis; IL-6, interleukin-6; MDA, malondialdehyde; TAS, total antioxidant status.

After adjustment, higher serum zonulin was associated with higher IL-6 (B = 0.982, β = 0.577, 95% CI 0.579 to 1.384; *P* < 0.001). The IL-6 model explained 46.3% of the observed variance (R^2^ = 0.463; adjusted R^2^ = 0.403; overall *P* < 0.001). Zonulin was also positively associated with MDA (B = 36.76, β = 0.597, 95% CI 25.32 to 48.20; *P* < 0.001), and the model explained 66.8% of the variance (R^2^ = 0.668; adjusted R^2^ = 0.631; overall *P* < 0.001). The association with TAS was inverse (B = −0.018, β = −0.345, 95% CI -0.032 to −0.003; *P* = 0.021), although the TAS model had modest explanatory power (R^2^ = 0.184; adjusted R^2^ = 0.092; overall *P* = 0.049). All predictor VIFs were < 5; the VIF for zonulin was 2.100 in each model.

## 4 Discussion

The principal finding was that serum zonulin was substantially higher in patients receiving maintenance HD than in healthy controls and was associated with higher IL-6 and MDA and lower TAS. These relationships persisted after adjustment for measured demographic and clinical covariates. The results therefore identify a coherent inflammatory-oxidative pattern associated with circulating zonulin in this cohort, while the cross-sectional design precludes conclusions about temporal sequence or causality.

Inflammation is a persistent feature of ESRD and is associated with cardiovascular complications, protein-energy wasting, endothelial dysfunction, immune disturbance, and mortality. Consistent with this background, IL-6 was markedly higher in the HD group. Previous studies have linked circulating IL-6 and related inflammatory markers to cardiovascular risk and adverse outcomes in dialysis populations,^9^, ^35^, ^36^ including progression of the malnutrition-inflammation-atherosclerosis phenotype.^37^ The adjusted association between zonulin and IL-6 suggests that the two markers covary beyond the measured effects of age, sex, diabetes, hypertension, smoking, albumin, hemoglobin, and phosphorus; it does not establish that barrier dysfunction initiated inflammatory activation.

The HD subjects demonstrated significantly higher MDA levels and lower TAS values as well, reflecting greater lipid peroxidation and reduced aggregate antioxidant status seen in kidney failure.^38^, ^39^, ^40^ Importantly, zonulin remained positively associated with MDA and inversely associated with TAS after adjustment. The association was strongest for MDA, whereas the TAS model explained comparatively little variance. This difference indicates that the measured covariates and zonulin captured only a limited proportion of the determinants of antioxidant status and cautions against treating TAS as a downstream consequence of zonulin.

Serum zonulin was markedly elevated in the HD group. This finding is consistent with reports of altered intestinal barrier-related markers in CKD and other renal disorders^29^, ^41^, ^42^ and with evidence of intestinal permeability abnormalities and gut-kidney crosstalk in glomerular disease and dialysis populations.^23^, ^43^ However, serum zonulin is an indirect and assay-dependent marker. Commercial immunoassays may recognize zonulin-family or other related proteins, and serum concentrations can diverge from direct functional permeability measurements.^24^, ^25^, ^26^, ^27^ Accordingly, the present result should be interpreted as an elevation in the immunoreactivity measured by the specified assay rather than definitive proof of intestinal hyperpermeability.

Several nonexclusive explanations could underlie the observed pattern. Uremia, dietary restrictions, gut dysbiosis, inflammation, oxidative stress, and altered protein metabolism may influence epithelial tight junctions or circulating zonulin-related proteins. Recent studies in maintenance HD have associated gut dysbiosis with immune alterations, metabolic disturbances, and dialysis-related complications.^44^, ^45^, ^46^, ^47^ These mechanisms provide biological plausibility, but the present study did not measure microbiota composition, endotoxin, bacterial DNA, gut-derived uremic toxins, or direct intestinal permeability and therefore cannot distinguish among them.

Experimental CKD models have shown reduced expression of tight-junction components, including occludin and claudin-4, together with increased permeability to bacterial toxins.^48^ Such findings support a possible mechanistic framework for gut-barrier disruption in kidney disease. They do not, however, demonstrate that the higher serum zonulin measured in this human cohort arose from the same pathway.

The positive associations of zonulin with IL-6 and MDA and its inverse association with TAS constitute the novel contribution of this study. They are compatible with coordinated gut-barrier, inflammatory, and oxidative disturbances but also with reverse or shared causation. Systemic inflammation and oxidative stress may themselves impair tight-junction integrity, whereas dietary factors, comorbidity, medication exposure, nutritional status, residual kidney function, or reduced clearance of circulating proteins may influence both zonulin immunoreactivity and the other biomarkers.^20^, ^34^, ^47^, ^49^, ^50^ Residual confounding therefore remains a substantive consideration despite multivariable adjustment.

From a translational perspective, these findings are hypothesis-generating. Serum zonulin cannot yet be recommended for screening, diagnosis, prognosis, treatment selection, or monitoring in HD. Its incremental value over established inflammatory markers such as C-reactive protein or IL-6 was not tested, and C-reactive protein was not measured in this study. Prospective studies should determine temporal relationships, within-patient reproducibility, responsiveness to interventions, assay standardization, and whether zonulin improves discrimination, calibration, or clinical decision-making beyond conventional biomarkers before any clinical role is proposed.

## 5 Study limitations

Several limitations should be considered. First, the cross-sectional design does not establish temporal order, directionality, or causation and cannot exclude reverse causation. Second, intestinal permeability was represented by a single serum zonulin immunoassay. Given concerns regarding the molecular specificity of commercial assays and the influence of diet, systemic inflammation, comorbidity, and other circulating proteins, the results should not be equated with a direct functional permeability measurement. Third, dialysis vintage, delivered dialysis dose or Kt/V, vascular access type, session frequency, cause of ESRD, residual kidney function, body mass index, detailed nutritional status, dietary patterns, and medication exposure (including anti-inflammatory or immunosuppressive agents, phosphate binders, vitamin D analogs, and erythropoiesis-stimulating agents) were not systematically available for analysis. These omissions may have produced residual confounding. Fourth, control eligibility was based on the available health history and exclusion criteria; the specific recruitment source and systematic screening for subclinical inflammatory or metabolic disorders were not retained in sufficient detail. Fifth, although Pearson coefficients were retained from the original analysis, complete archived diagnostics for linearity and influential observations were unavailable during revision; these correlations should therefore be interpreted cautiously and verified from the raw data before final submission if those data are available. Sixth, the absence of C-reactive protein and of direct or complementary gut-barrier measures, including intestinal permeability tests, endotoxin, bacterial DNA, gut-derived uremic toxins, and microbiome profiling, limited biological and clinical interpretation. Finally, the single-center setting may restrict generalizability.

The study also has strengths. It included equally sized, age- and sex-comparable groups; standardized predialysis sampling; simultaneous assessment of a putative intestinal-barrier marker, an inflammatory cytokine, lipid peroxidation, and antioxidant status; and adjusted analyses with effect estimates and 95% CIs. These features improve the internal coherence of the observed associations but do not remove the limitations of biomarker specificity, unmeasured confounding, or the cross-sectional design.

## 6 Conclusion

Patients receiving maintenance HD had higher serum zonulin, IL-6, and MDA concentrations and lower TAS than healthy controls. Within the HD group, serum zonulin was independently associated with higher IL-6 and MDA and lower TAS after adjustment for selected demographic and clinical covariates. These findings support an association between circulating zonulin immunoreactivity and inflammatory-oxidative imbalance, but they neither establish intestinal hyperpermeability as the cause of that imbalance nor validate zonulin for clinical use. Multicentre longitudinal studies that include the measurement of permeability parameters, gut flora assessment, gut-produced toxins, dialysis-related parameters, drug exposure, and classic inflammatory markers are needed to establish the order of events and the predictive capacity of these biomarkers.

## CRediT authorship contribution statement

**Ali B. Roomi:** Writing – review & editing, Writing – original draft, Methodology, Formal analysis. **Hussein Jawad Kadhim:** Writing – review & editing, Writing – original draft, Supervision, Methodology, Investigation, Formal analysis, Data curation. **Abbas Khairi Fadhil:** Writing – review & editing, Writing – original draft, Supervision, Methodology, Investigation, Formal analysis, Data curation. **Ibrahim Abdullah Hamzah:** Writing – review & editing, Writing – original draft, Supervision, Methodology, Investigation, Formal analysis, Data curation.

## Ethics approval and consent to participate

The study design was reviewed and approved by the Ethics Committee, Department of Biochemistry, College of Medicine, University of Thi-Qar, Nasiriyah, Iraq (IRB Approval No. 05/2025), as well as the Directorate General of Health, Thi-Qar Governorate, Nasiriyah, Iraq. All procedures were carried out in compliance with the principles of the Declaration of Helsinki. Written consent was obtained from all subjects prior to their participation in the study.

## Ethics declaration

Written informed consent to take part in the study and to publish the article has been obtained from all participants or their legal representatives. The privacy rights of participants have been observed.

This study was performed in compliance with relevant laws, regulatory frameworks and guidelines where the research took place. This study was approved by the IRB Approval No. 05/2025. (Approval No. IRB Approval No. 05/2025)

## Funding

This research received no external funding.

## Declaration of competing interest

The authors declare the following financial interests/personal relationships which may be considered as potential competing interests: Ali B. Roomi reports was provided by University of Thi-Qar. Ali B. Roomi reports a relationship with University of Thi-Qar that includes:. If there are other authors, they declare that they have no known competing financial interests or personal relationships that could have appeared to influence the work reported in this paper.

## References

[1] GBD 2023 Chronic Kidney Disease Collaborators (2025). Global, regional, and national burden of chronic kidney disease in adults, 1990-2023, and its attributable risk factors: a systematic analysis for the global burden of disease study 2023. Lancet.

[2] Yan Z., Shao T. (2024). Chronic inflammation in chronic kidney disease. Nephron.

[3] Chen J. (2024). Inflammatory burden index: a superior prognostic biomarker of systemic inflammation in patients on peritoneal dialysis. J Inflamm Res.

[4] Pethő Á.G. (2025). Increased cardiovascular mortality in hemodialysis: the role of chronic inflammation, complement activation, and non-biocompatibility. Toxins.

[5] Kadatane S.P. (2023). The role of inflammation in CKD. Cells.

[6] V. Kalacun et al., “Oxidative stress and inflammation in hemodialysis: a comparison of patients with or without advanced nonalcoholic fatty liver disease (NAFLD),” Ren Fail, vol. 47, no. 1, Art. no. 2455523, 2025, doi:10.1080/0886022X.2025.2455523. PMC11755732 39842820

[7] Edam K.A., Roomi A.B. (2024). The role of titanium dioxide nanoparticles on the effectiveness of erythropoietin alfa drug in rats with anemia accompanied with chronic kidney disease. BioNanoScience.

[8] Ji H. (2023). Advances in enhancing hemocompatibility of hemodialysis hollow-fiber membranes. Adv Fiber Mater.

[9] O. Istanbuly, J. Belcher, M. Tabinor, I. Solis-Trapala, M. Lambie, and S. J. Davies, “Estimating the association between systemic Interleukin-6 and mortality in the dialysis population: re-analysis of the GLOBAL fluid study, systematic review and meta-analysis,” BMC Nephrol, vol. 24, Art. no. 312, 2023, doi:10.1186/s12882-023-03370-4. PMC10601265 37884903

[10] Qureshi R. (2023). Interleukin-6-to-albumin ratio as a superior predictor of mortality in end stage kidney disease patients. Am J Nephrol.

[11] C. C. Rusu, C. C. Ciucanu, D. R. S. Ștefan, E. E. V. M. Popa, A. R. Covic, and A. Burlacu, “Exploring the associations between inflammatory biomarkers, nutritional biomarkers, cardiovascular events, and survival in chronic hemodialysis patients,” *J Clin Med*, vol. 14, no. 4, Art. no. 1139, 2025, doi:10.3390/jcm14041139. PMC11855970 40004669

[12] L. Zhang, F. Xu, and L. Hou, “IL-6 and diabetic kidney disease,” Front Immunol, vol. 15, Art. no. 1465625, 2024, doi:10.3389/fimmu.2024.1465625. PMC11693507 39749325

[13] H.-J. Ho and H. Shirakawa, “Oxidative stress and mitochondrial dysfunction in chronic kidney disease,” Cells, vol. 12, no. 1, Art. no. 88, 2023, doi:10.3390/cells12010088. PMC9818928 36611880

[14] Kishi S., Nagasu H., Kidokoro K., Kashihara N. (2024). Oxidative stress and the role of redox signalling in chronic kidney disease. Nat Rev Nephrol.

[15] P. Bergin, A. Leggett, C. R. Cardwell, J. V. Woodside, A. Thakkinstian, A. P. Maxwell, and G. J. McKay, “The effects of vitamin E supplementation on malondialdehyde as a biomarker of oxidative stress in haemodialysis patients: a systematic review and meta-analysis,” BMC Nephrol, vol. 22, Art. no. 126, 2021, doi:10.1186/s12882-021-02328-8. PMC8034191 33832458

[16] O. Y. A. Lee et al., “Potentials of natural antioxidants in reducing inflammation and oxidative stress in chronic kidney disease,” *Antioxidants (Basel)*, vol. 13, no. 6, Art. no. 751, 2024, doi:10.3390/antiox13060751. PMC11201162 38929190

[17] Evenepoel P., Stenvinkel P., Shanahan C., Pacifici R. (2023). Inflammation and gut dysbiosis as drivers of CKD–MBD. Nat Rev Nephrol.

[18] Biruete A., Jeong J.H., Barnes J.L., Wilund K.R. (2024). Feeling gutted in chronic kidney disease (CKD): gastrointestinal disorders and therapies to improve gastrointestinal health in individuals with CKD, including those undergoing dialysis. Semin Dial.

[19] Chao Y.T., Lin Y.K., Chen L.K., Huang P., Hsu Y.C. (2023). Role of the gut microbiota and their metabolites in hemodialysis patients. Int J Med Sci.

[20] G.-A. Georgopoulou et al., “Altered expression of intestinal tight junctions in patients with chronic kidney disease: a pathogenetic mechanism of intestinal hyperpermeability,” Biomedicines, vol. 12, no. 2, Art. no. 368, 2024, doi:10.3390/biomedicines12020368. PMC10887073 38397970

[21] X. Lim, L. Ooi, U. Ding, H. H. L. Wu, and R. Chinnadurai, “Gut microbiota in patients receiving dialysis: a review,” *Pathogens* (Basel, Switzerland), vol. 13, no. 9, Art. no. 801, 2024, doi:10.3390/pathogens13090801. PMC11434973 39338992

[22] K. Tsuji, N. Uchida, H. Nakanoh, K. Fukushima, S. Haraguchi, S. Kitamura, and J. Wada, "The gut-kidney axis in chronic kidney diseases," Diagnostics (Basel), vol. 15, no. 1, Art. no. 21, 2025, doi:10.3390/diagnostics15010021. PMC11719742 39795549

[23] J. Du, X. Zhang, Q. Ren, H. Wang, X. Wang, C. Song, Y. Dai, X. Dai, Q. Hu, D. Zhang, and H. Zhu, “The role of the gut microbiota in complications among hemodialysis patients,” Microorganisms, vol. 12, no. 9, Art. no. 1878, 2024, doi:10.3390/microorganisms12091878. PMC11434415 39338552

[24] Mohammadi-Kordkhayli M. (2025). Elucidating the significance of zonulin in the pathogenesis of chronic inflammatory disorders: emphasis on intestinal barrier function and tight junction regulation. Curr Med Chem.

[25] Yu J., Shen Y., Zhou N. (2021). Advances in the role and mechanism of zonulin pathway in kidney diseases. Int Urol Nephrol.

[26] L. Scheffler et al., "Widely used commercial ELISA does not detect precursor of haptoglobin2, but recognizes properdin as a potential second member of the zonulin family," Front Endocrinol, vol. 9, Art. no. 22, 2018, doi:10.3389/fendo.2018.00022. PMC5807381 29459849

[27] M. Ajamian, D. Steer, G. Rosella, and P. R. Gibson, "Serum zonulin as a marker of intestinal mucosal barrier function: may not be what it seems," PLoS One, vol. 14, no. 1, Art. no. e0210728, 2019, doi:10.1371/journal.pone.0210728. PMC6331146 30640940

[28] A. Górecka, A. Jura-Półtorak, E. M. Koźma, A. Szeremeta, K. Olczyk, and K. Komosińska-Vassev, “Biochemical modulators of tight junctions (TJs): occludin, claudin-2 and zonulin as biomarkers of intestinal barrier leakage in the diagnosis and assessment of inflammatory bowel disease progression,” *Molecules* (Basel, Switzerland), vol. 29, no. 19, Art. no. 4577, 2024, doi:10.3390/molecules29194577. PMC11478261 39407507

[29] B. Macura, A. Kiecka, and M. Szczepanik, “Intestinal permeability disturbances: causes, diseases and therapy,” Clin Exp Med, vol. 24, no. 1, Art. no. 232, 2024, doi:10.1007/s10238-024-01496-9. PMC11438725 39340718

[30] A. Kirik, B. Yılmaz, M. H. Sipahioğlu, H. N. Sipahioğlu, İ. Bilgiçler, E. Parlak, T. Mutlu, Ö. Kama Başcı, and Ö. Toprak, “The role of circulating zonulin levels in patients with chronic kidney disease stages 3 to 5 not on dialysis,” Medicine (Baltimore), vol. 104, no. 21, Art. no. e42185, 2025, doi:10.1097/MD.0000000000042185. PMC12114037 40419894

[31] Li Q., Du X., Wang Y., Liu J., Zhang H. (2023). Zonulin, as a marker of intestinal permeability, is elevated in IgA nephropathy and IgA vasculitis with nephritis. Clin Kidney J.

[32] Pohjonen J., Kaukinen K., Huhtala H. (2024). Indirect markers of intestinal damage in IgA nephropathy. Nephron.

[33] Y. Fan, S. Pedersen, and J. M. Andersen, “Advancements in understanding the role of intestinal barrier dysfunction in IgA nephropathy: a focus on gut–kidney crosstalk,” BMC Nephrol, vol. 25, Art. no. 286, 2024, doi:10.1186/s12882-024-03646-3.

[34] Q. Wang, Y. Han, L. Pang, Z. Zhou, and L. Dai, "Gut microbiome remodeling in chronic kidney disease: implications of kidney replacement therapies and therapeutic interventions," Front Med, vol. 12, Art. no. 1620247, 2025, doi:10.3389/fmed.2025.1620247. PMC12303993 40735439

[35] Chertow G.M. (2024). IL-6 inhibition with clazakizumab in patients receiving maintenance dialysis: a randomized phase 2b trial. Nat Med.

[36] F. Guerrero et al., "New biomarkers of inflammation associated with haemodialysis," Clin Kidney J, vol. 18, no. 8, Art. no. sfaf223, 2025, doi:10.1093/ckj/sfaf223. PMC12358798 40832121

[37] S. Chen et al., “Inflammatory markers predict MIA syndrome progression and cardiovascular disease outcomes in maintenance dialysis patients,” BMC Nephrol, vol. 26, Art. no. 654, 2025, doi:10.1186/s12882-025-04578-2. PMC12629059 41257631

[38] A. Tsinari et al., “The clinical utility and plausibility of oxidative and antioxidant variables in chronic and end-stage kidney disease: a review of the literature,” Int J Mol Sci, vol. 26, no. 7, Art. no. 3376, 2025, doi:10.3390/ijms26073376. PMC11989862 40244241

[39] M. B. Marques et al., “Assessment of the antioxidant status and markers of oxidative stress in patients with kidney failure: effects of a hemodialysis session,” An Acad Bras Cienc, vol. 96, suppl. 1, p. e20240297, 2024, doi:10.1590/0001-3765202420240297. 39319866

[40] Ebert T., Neytchev O., Witasp A., Kublickiene K., Stenvinkel P., Shiels P.G. (2021). Inflammation and oxidative stress in chronic kidney disease and dialysis patients. Antioxid Redox Signal.

[41] Zadora W., Jacobs E., Lauriola M. (2026). Intestinal barrier dysfunction in chronic kidney disease: evidence, mechanisms, and its potential clinical implications. Nephrology Dialysis Transplantation.

[42] Suliman L. (2025). Gut microbiome in patients with chronic kidney disease stages 4 and 5: a systematic literature review. Int J Mol Sci.

[43] Seikrit C. (2023). Intestinal permeability in patients with IgA nephropathy and other glomerular diseases: an observational study. J. Nephrol..

[44] Y. Lv et al., “Immune-microbiota dysregulation in maintenance hemodialysis: a 16S rRNA sequencing-based analysis of gut flora and T cell profiles,” Ren Fail, vol. 47, no. 1, Art. no. 2498630, 2025, doi:10.1080/0886022X.2025.2498630. PMC12082729 40375064

[45] V. Lazarevic, D. Teta, M. Pruijm, C. Saffon, N. Malhamé, et al., “Gut microbiota associations with chronic kidney disease: insights into nutritional and inflammatory parameters,” Front Microbiol, vol. 15, Art. no. 1298432, 2024, doi:10.3389/fmicb.2024.1298432. PMC11148242 38835485

[46] Wu H.-Y. (2023). Microbiota analysis in the hemodialysis population—focusing on Enterobacteriaceae. J Microbiol Immunol Infect.

[47] A. Matar, S. Al-Ani, and M. Y. Kelleni, “Intestinal barrier impairment, preservation, and repair: an update,” Nutrients, vol. 16, no. 20, Art. no. 3494, 2024, doi:10.3390/nu16203494. PMC11509958 39458489

[48] M. de la Luz Villela-Torres, M.-C. Prado-Uribe, M. Ávila Díaz, H. Quezada Pablo, E. Soria-Castro, N. Esturau Escofet, C. E. Flores Maldonado, and R. Paniagua, “Effect of high sodium intake on gut tight junctions' structure and permeability to bacterial toxins in a rat model of chronic kidney disease,” Arch Med Res, vol. 55, no. 3, Art. no. 102969, 2024, doi:10.1016/j.arcmed.2024.102969. 38484487

[49] E. Coll, S. López-Suñé, M. Massana, E. Diago-Cabezudo, M. Flores-Mateo, and M. Betriu, “Gut dysbiosis and its role in the anemia of chronic kidney disease,” Toxins, vol. 16, no. 11, Art. no. 495, 2024, doi:10.3390/toxins16110495. PMC11598790 39591250

[50] C. Li, J. K.-C. Ng, G. C.-K. Chan, W. W.-S. Fung, K.-B. Lai, P. Y.-K. Poon, C. C.-W. Luk, K.-M. Chow, and C.-C. Szeto, “Gut permeability, circulating bacterial fragments and measures of congestion in peritoneal dialysis,” Clin Kidney J, vol. 17, no. 3, Art. no. sfae056, 2024, doi:10.1093/ckj/sfae056. PMC10956420 38516523

